# Correlations of Obstructive Sleep Apnea Syndrome and Daytime Sleepiness with the Risk of Car Accidents in Adult Working Population: A Systematic Review and Meta-Analysis with a Gender-Based Approach

**DOI:** 10.3390/jcm11143971

**Published:** 2022-07-08

**Authors:** Valeria Luzzi, Marta Mazur, Mariana Guaragna, Gabriele Di Carlo, Luisa Cotticelli, Giuseppe Magliulo, Beatrice Marasca, Valentina Pirro, Gianni Di Giorgio, Artnora Ndokaj, Patrizio Pasqualetti, Ilaria Simonelli, Agnese Martini, Emma Pietrafesa, Antonella Polimeni

**Affiliations:** 1 Department of Oral and Maxillofacial Sciences, Sapienza University of Rome, Via Caserta 6, 00161 Rome, Italy; valeria.luzzi@uniroma1.it (V.L.); mariana.guaragna@uniroma1.it (M.G.); gabriele.dicarlo@uniroma1.it (G.D.C.); luisa.cotticelli@gmail.com (L.C.); beatrice.marasca@uniroma1.it (B.M.); valentina.pirro@uniroma1.it (V.P.); gianni.digiorgio@uniroma1.it (G.D.G.); artnora.ndokaj@uniroma1.it (A.N.); antonella.polimeni@uniroma1.it (A.P.); 2 Department of Sensory Organs, Sapienza University of Rome, 00100 Rome, Italy; giuseppe.magliulo@uniroma1.it; 3 Department of Public Health and Infectious Diseases, Section of Medical Statistics, Sapienza University of Rome, 00100 Rome, Italy; patrizio.pasqualetti@uniroma1.it; 4 Service of Medical Statistics and Information Technology, Fatebenefratelli Foundation for Health Research and Education, 00100 Rome, Italy; ilaria.simonelli@afar.it; 5 Department of Medicine, Epidemiology, Occupational and Environmental Hygiene, INAIL, Via Fontana Candida 1, Monte Porzio Catone, 00078 Rome, Italy; a.martini@inail.it (A.M.); e.pietrafesa@inail.it (E.P.)

**Keywords:** sleep apnea, obstructive sleep apnea syndrome, OSAS, day-time sleepiness, sleep-disordered breathing, driving, motor vehicle crashes, car accidents, safety, driving safety, systematic review, meta-analysis

## Abstract

Obstructive sleep apnea syndrome (OSAS) is an under-recognized clinical condition and is correlated with sleepiness and impaired cognitive function. Objectives: The primary aim of this systematic review, developed within the Sleep@OSA project, was to determine the correlations of obstructive sleep apnea syndrome, daytime sleepiness and sleep-disordered breathing with the risk of car accidents in adult working populations; a secondary aim was to analyze the epidemiologic data with a gender-based approach to identify differences between women and men in the data and in associated risk factors. Methods: Clinical trials and studies reporting data on the frequency of car accidents involving adult working population with daytime sleepiness and/or OSAS compared with a control group of participants were included. Literature searches of free text and MeSH terms were performed using PubMed, Google Scholar, the Cochrane Library and Scopus from 1952 to 3 May 2021. Results and Conclusions: The search strategy identified 2138 potential articles. Of these, 49 papers were included in the qualitative synthesis, and 30 were included in the meta-analysis. Compared with controls, the odds of car accidents were found to be more than double in subjects with OSAS (OR = 2.36; 95% CI 1.92–2.91; *p* < 0.001), with a similar risk between commercial motor vehicle drivers (OR = 2.80; 95% CI 1.82–4.31) and noncommercial motor vehicle drivers (OR = 2.32; 95% CI 1.84–2.34). No significant correlation was found between sleepiness and car crashes, but subjects with sleep-disordered breathing were at increased risk of car accidents (OR = 1.81; 95% CI 1.42–2.31; *p* < 0.001). To our surprise, although epidemiological studies on the risk of road accidents in the adult population with OSAS and daytime sleepiness are currently very abundant, specific data on the female population are not available.

## 1. Introduction

Motor vehicle crash risk can be attributed to sleepiness and impaired vigilance conditions [1]. Obstructive sleep apnea syndrome (OSAS) is an under-recognized clinical condition and is correlated with sleepiness and impaired cognitive function [1,2,3,4,5,6,7,8].

According to the American Academy of Sleep Medicine guidelines, the evaluation of risk factors correlated with OSAS requires a clinical judgement based on an integrated assessment of history, symptoms and physical and clinical findings [9]. Medical history that suggests increased risk of OSAS includes hypertension requiring more than two medications for control or refractory hypertension, type 2 diabetes, atrial fibrillation or nocturnal dysrhythmias, congestive heart failure, stroke, pulmonary hypertension, motor vehicle accidents (especially those associated with sleepiness/drowsiness) and being under consideration for bariatric surgery. Symptoms that indicate an increased risk of OSAS include snoring, daytime sleepiness, witnessed apneas, complaints of awakening with sensation of gasping or choking, non-refreshing sleep, frequent awakening (sleep fragmentation) or difficulty staying asleep (maintenance insomnia), morning headaches, decreased concentration, problems or difficulty with memory or memory loss and irritability. Physical/clinical findings that suggest increased risk of OSAS include a high score on an OSAS screening questionnaire (e.g., Berlin, Epworth), increased neck circumference (>17 inches in men, >16 inches in women), a modified Mallampati score of 3 or 4 (assessment of the oral cavity), retrognathia, lateral peritonsillar narrowing, macroglossia, tonsillar hypertrophy, elongated/enlarged uvula, high arched/narrow hard palate, nasal abnormalities such as polyps, deviation and turbinate hypertrophy, and obesity [9,10].

Studies report that the burden of undiagnosed OSAS is high and that drowsy driving is the major cause of both highway and fatal crashes. Moreover, OSAS is significantly correlated with excessive daytime sleepiness [10].

The Report of the Obstructive Sleep Apnoea Working Group, based on European Committee Directive 2006/126/EC, proposed adding OSAS as a clinical condition to Annex III, the list of all the diseases linked to driving risk [11].

Moreover, as OSAS prevalence is also increasing as a result of the obesity pandemic, a current gold standard for OSAS diagnosis based on PSG is under-developed relative to the prevalence of the condition. There is a need for a validated and cost-effective diagnostic tool to rapidly identify cases suspected for OSAS as the risk of motor vehicle crashes is removed when OSAS subjects are treated with continuous positive airway pressure (CPAP) [12].

To date, there is a lack of data on the prevalence of OSAS in a matched male and female population with comparable physical and medical characteristics. Several studies reported a two- to threefold greater risk of OSAS in men compared with women, but these findings were based on: (i) a narrative report published in 1996 [13]; (ii) a clinical study conducted in 2005 using a matched-pair approach on a sample population of 40 pairs of patients over the age of 65 [14]; (iii) gender differences explained by dissimilar body fat distribution, disparities in pharyngeal anatomy and hormonal variances that act on upper airway muscles and cause its collapse [15]; and (iv) a community clinical trial conducted in the late 1990s on a small sample of Hong Kong-only women [16]. In addition, the great majority of epidemiological studies were focused on professional drivers, who are primarily adult males [5].

On the other side, a clinical study of 1166 subjects aiming to assess differences between men and women referred to a sleep unit for symptoms of OSAS, with a male/female ratio of 4.9:1, showed that the frequency of snoring and daytime sleepiness was similar in the two genders, while fatigue, morning headaches, insomnia, depression and use of sedatives were more frequent in women. The authors concluded that it is likely that women with OSAS may be underdiagnosed due to sociocultural factors [17,18]. To enhance the variability and reduce the ambiguity of the epidemiological data currently available, we cite the results of a recent study of 1208 men and women in northeastern Germany, where the OSAS prevalence was 59% and 33% for apnea hypopnea index ≥ 5 (AHI) and 30% and 13% for AHI ≥ 15, for men and women, respectively [19].

Given such clinical scenarios, primary aim of this systematic review was to determine the correlations of obstructive sleep apnea syndrome and daytime sleepiness with the risk of car accidents in adult working populations; a secondary aim was to analyze the epidemiologic data with a gender-based approach in an attempt to define risk factors associated specifically with women versus men.

## 2. Materials and Methods

This systematic review was conducted in accordance with the Preferred Reporting Items for Systematic Reviews and Meta-Analyses (PRISMA) statement and the guidelines from the Cochrane Handbook for Systematic Reviews of Interventions [20]. The study protocol was registered after the screening stage (PROSPERO CRD42021262932).

### 2.1. Eligibility Criteria

The following inclusion criteria were applied for this systematic review: (a) randomized controlled trials (RCT); (b) clinical trials; (c) cohort studies; (d) cross-sectional studies; (e) case-control studies; (f) pilot studies; (g) prospective and observational studies; (h) retrospective studies; (i) clinical human studies based on human dentate and (g) studies published in English, French, German, Spanish, Polish or Albanian. The broad inclusion criteria aimed to be as sensitive as possible. Studies on the frequency of car accidents involving adult working populations (age 18 or older) with OSAS diagnosed using either polysomnography (PSG), i.e., full, multiparametric test, or standardized questionnaires and compared with a control group of subjects not affected by OSAS were also included. The following were the exclusion criteria: (a) studies based on driving simulators; (b) studies analyzing the prevalence of sleep disorders without reference to driving accidents; (c) studies on subjects with other sleep disorders or with medical or other conditions or who consumed drugs, alcohol, or other substances that provoke daytime sleepiness; (d) studies lacking effective statistical analysis or (e) abstracts and author debates or editorials. Systematic reviews and meta-analyses were also excluded.

### 2.2. Search Strategy and Study Selection

Literature searches of free text and MeSH terms were performed using PubMed, Google Scholar, Cochrane Library and Scopus from 1952 to 3 May 2021. All searches were conducted using a combination of subject headings and free-text terms. The final search strategy was determined through several pre-searches. The keywords used in the search strategy were as follows:

1. Pubmed: ((“working population” OR “working adults” OR “working class” OR “workers”) AND (“sleep apnea syndrome” OR “apnea” OR “obstructive sleep apnea”) AND (“accidents” OR “safety” OR “injuries”)).
2. Scopus: TITLE-ABS-KEY ((working population OR working adults OR working class OR workers) AND (sleep apnea syndrome OR apnea OR obstructive sleep apnea) AND (accidents OR safety OR injuries)).
3. Google Scholar: “working adults” AND “OSAS” AND “working accidents”.

The titles and abstracts of the identified studies were screened independently by two calibrated reviewers (MM, GDC). Calibration with regards to possible eligibility and inclusion of studies was performed on a subset of 20 studies prior to searching all databases. Based on the abstracts, a consensus decision was made about reading the full text; the full text was read when there was any doubt. Disagreements about the final selection were resolved by consensus or, in cases of continuing disagreement, through consultation with a third reviewer (VL). The reviewers also recorded and compared their reasons for excluding studies, and a consensus was reached when there were disagreements. To determine the degree of agreement in the selection of abstracts and full papers, the kappa index of inter-observer agreement was calculated using the tool accessible at the following website: http://faculty.vassar.edu/lowry/kappa.html (accessed on 13 January 2022). Only studies fulfilling all the inclusion criteria were included in both the qualitative and quantitative synthesis. The agreement rate was 95%.

### 2.3. Data Collection

For each eligible study, data were independently extracted by two authors (M.M. and G.D.C.) and examined by the third author (V.L.) by creating a piloted and independently categorized spreadsheet dataset in accordance with the Cochrane Collaboration guidelines. In cases of missing data, MM contacted the corresponding author of the related research via email and excluded those for which no reply was received.

### 2.4. Data Items

The following data items were recorded: author; year; study type (cohort, case study, longitudinal or cross-sectional); country; population (number of subjects and mean age and/or SD); percentage of female subjects; study setting (hospital, private practice, university clinic); recruitment of subjects (professional or nonprofessional drivers); neck circumference; body mass index (BMI); number of cases (car crashes and car accidents in subjects with OSAS; car crashes in subjects with ESS ≥ 10 and subjects with sleep-disordered breathing, SDB); number of controls (car crashes in subjects without OSAS; non-car accidents in subjects with OSAS; car crashes in subjects without ESS ≥ 10 and subjects without SDB); percentage of female subjects in both groups; if present, diagnosis of OSAS, severity of OSAS and how OSAS was assessed; oxygen saturation (%); sleepiness and how it was assessed; driving exposure (hours/week and/or km driven/year); definition of crash and period of time assessed for crashes. Subjects with OSAS are considered patients who were diagnosed instrumentally using PSG.

### 2.5. Quality Assessment and Risk of Bias

According to the PRISMA statements, the evaluation of the methodological quality gives an indication of the strength of evidence provided by the study because methodological flaws can result in biases. For cross-sectional, case-control and cohort studies, according to the Newcastle–Ottawa scale (NOS), the possible quality assessment score ranges from zero to nine points, with a high score indicating good study quality.

Selection bias (retained allocation concealment), performance and detection bias (blinding of participants and operators), attrition bias (patient dropout, wash-out period of crossover trials, missing values or participants, too short duration of follow-up) and reporting bias (selective reporting, unclear eliminations, missing results) were recorded, evaluated and allocated according to Cochrane guidelines [20].

### 2.6. Outcomes

The definitions of cases and controls were different among the included studies: several meta-analyses were performed by pooling together the studies with the same definition.

Outcomes of interest included the following definitions of cases:

(i) car crashes in subjects with OSAS and total number of subjects with OSAS
(ii) car accidents in subjects with OSAS and total number of car accidents, both of which were analyzed depending on the different types of drivers (CMVD, commercial motor vehicle drivers and NCMVD, noncommercial motor vehicle drivers)
(iii) car crashes in subjects with ESS ≥ 10 and total subjects with ESS ≥ 10
(iv) car crashes in subjects with SDB and total subjects with SDB.

Definition of controls:

(i) car crashes in subjects without OSAS and total number of subjects without OSAS
(ii) non-car accidents in subjects with OSAS and total number of non-car accidents, both of which were analyzed depending on the different types of drivers (CMVD, commercial motor vehicle drivers and NCMVD, non-commercial motor vehicle drivers)
(iii) car crashes in subjects without ESS ≥ 10 and total subjects without ESS ≥ 10
(iv) car crashes in subjects without SDB and total subjects without SDB.

Studies with overlapping populations were excluded, and only those with the most complete and recent data were selected.

### 2.7. Meta-Analysis

A random-effect model to combine the data into a pooled odds ratio (OR) was performed. Continuity correction was applied if there was a zero cell.

The heterogeneity was investigated using the chi-squared test and quantified inconsistency with I^2^. This index describes the percentage of the variability in effect estimates that is due to heterogeneity rather than sampling error, ranging from 0 (no heterogeneity) to 100 (maximum heterogeneity). This index was interpreted using the classification proposed by Higgins et al. [18]: 0% = no heterogeneity; 25% = low heterogeneity; 50% = moderate heterogeneity and 75% = high heterogeneity. OT explains the heterogeneity, and subgroup analysis was performed.

Contour-enhanced funnel plots were used to investigate publication bias including contours of statistical significance. If studies appear to be missing in areas of low statistical significance, then the asymmetry could be due to publication bias. Missing studies in areas of high statistical significance are less likely to be caused by publication bias. If there were enough studies (minimum of 10 studies), Begg and Mazumdar’s test was then used to provide statistical evidence for funnel plot symmetry [21]. Separate subgroup meta-analyses were performed based on the scale used in each study, on study design and on the different types of drivers (CMVD, commercial motor vehicle drivers and NCMVD, non-commercial motor vehicle drivers).

A *p* value < 0.05 was considered statistically significant. All analyses were performed with R.

## 3. Results

### 3.1. Study Selection

The search strategy identified 2138 potential articles: 367 from PubMed, 36 from Scopus, 1700 from Google Scholar and 35 from manual search. After the removal of duplicates, 1752 articles were analyzed.

**Figure 1:**
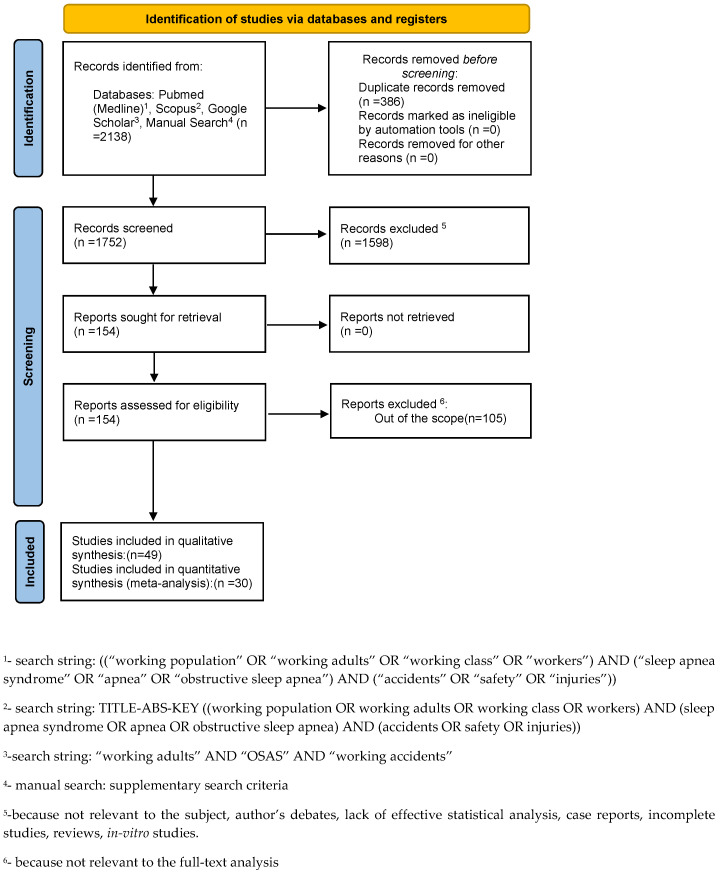
The flow chart of the search.

Subsequently, 1598 papers were excluded because they did not meet the inclusion criteria. Of the remaining 154 papers, 105 were excluded because they were not relevant to the subject of the study. The remaining 49 papers were included in the qualitative synthesis, and 30 of these were included in the meta-analysis (Figure 1). Table S1 summarizes the characteristics of each of the 49 included studies. All the included papers reported ORs for the study’s relevant query data.

### 3.2. Quality Assessment and Risk of Bias

The tools we used to determine the risk of bias and the quality of the studies included were based on the NOS, which are widely used for observational studies.

According to the NOS on cross-sectional (n:17) [22,23,24,25,26,27,28,29,30,31,32,33,34,35,36,37,38]; case–control (n:25) [39,40,41,42,43,44,45,46,47,48,49,50,51,52,53,54,55,56,57,58,59,60,61,62,63] and cohort studies (n:7) [64,65,66,67,68,69,70], the authors evaluated the qualities and the risk of bias of all included studies based on object selection, comparability and exposure [71]. A star was described as an appropriate entry, with each star representing one point. The possible quality assessment score ranged from zero to nine points, with a high score indicating good quality study. In the evaluation of the quality of cross-sectional studies, the total scores of 4 studies were lower than or equal to 5, indicating low-quality studies; the total scores of the other 13 were 6 or higher, indicating medium- or high-quality studies (Table 1). For the case–control studies, the total scores of 12 studies were lower or equal to 5, indicating low-quality studies, and the total scores of the other 13 were 6 or higher, indicating medium- or high-quality studies (Table 2). Regarding the quality of the cohort studies, the total scores for all included studies except two were lower than or equal to 5, indicating low-quality studies (Table 3). This categorization of quality and risk was used for descriptive purposes, not for statistical evaluation.

### 3.3. Study Populations

From each study, we extracted the outcomes of interest.

Cases:

(i) 872 car crashes in subjects with OSAS and 6873 subjects with OSAS [24,25,40,41,43,45,46,47,48,50,52,53,65,70]
(ii) 1000 car accidents in subjects with OSAS and 2964 car accidents [22,28,42,49,51,54,55,59,60,61]
(iii) 1233 car crashes in subjects with ESS ≥ 10 and 3208 subjects with ESS ≥ 10 [27,31,37,58]
(iv) 311 car crashes in subjects with SDB and 513 subjects with SDB [33,57].

Controls:

(i) 538 car crashes in subjects without OSAS and 7914 subjects without OSAS [24,25,40,41,43,45,46,47,48,50,52,53,65,70]
(ii) 2093 non-car accident in subjects with OSAS and 5328 non-car accidents [22,28,42,49,51,54,55,59,60,61]
(iii) 3000 car crashes in subjects without ESS ≥ 10 and 8517 subjects without ESS ≥ 10 [27,31,37,58]
(iv) 264 car crashes in subjects without SDB and 573 subjects without SDB [33,57].

### 3.4. Meta-Analysis

#### 3.4.1. Car Crashes in Subjects with OSAS and Total Number of Subjects with OSAS

Data were extracted from 14 studies [24,25,40,41,43,45,46,47,48,50,52,53,65,70], and there was a total of 872 car crashes with OSAS over a total of 6873 total OSAS. There were also 538 car crashes without OSAS over 7914 total subjects without OSAS.

The pooled OR was equal to 2.36 (95% CI 1.92–2.91; *p* < 0.001), indicating that the probability of car crash with OSAS was 2.36 times that without OSAS. The heterogeneity between studies was moderate (I^2^ = 46%, *p* = 0.03).

**Figure 2:**
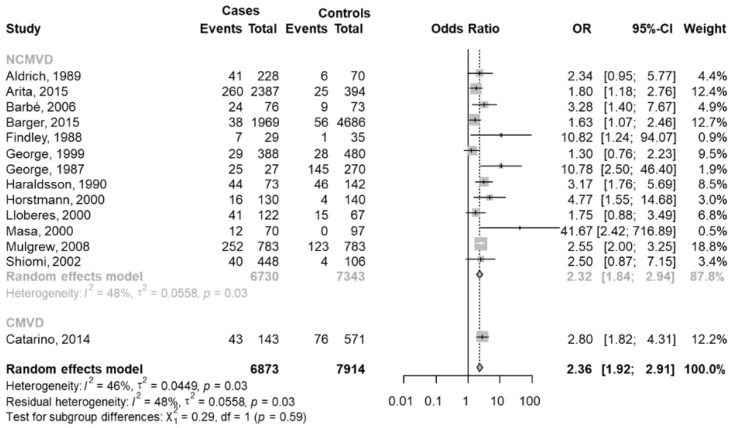
The forest plot of the subgroup meta-analysis of car crashes with and without OSAS based on types of drivers [24,25,40,41,43,45,46,47,48,50,52,53,65,70]. Events Cases are defined as car crashes with OSAS (total of 872) and Total Cases as subjects with OSAS (6873); Events Controls are defined as car crashes without OSAS (total 538) and Total Controls as subjects without OSAS (7914). In each group, a diamond represents the pooled OR estimate based on the random effects model.

A subgroup meta-analysis was performed based on the types of drivers. Only one study [25] considered the CMVD drivers. In the group of NCMVD drivers [24,40,41,43,45,46,47,48,50,52,53,65,70], the pooled OR was equal to 2.32 (95% CI 1.84–2.34), a significant effect in line with the overall meta-analysis (Figure 2).

**Figure 3:**
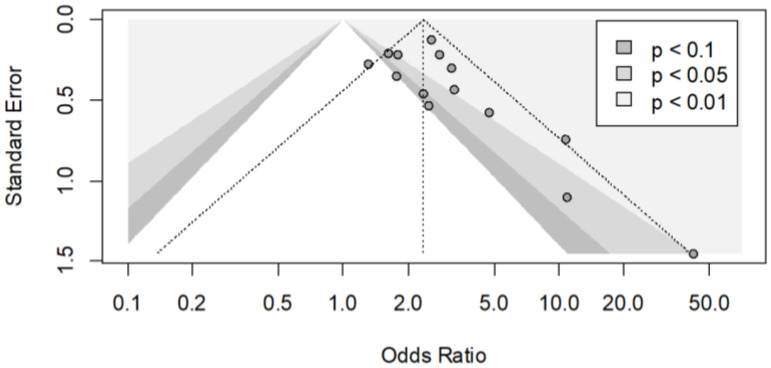
The contour funnel plot of meta-analyses of car crashes with and without OSAS, with contours of statistical significance at 1%, 5% and 10%. It is helpful to investigate if in a meta-analysis, some studies could have been suppressed based on their statistical significance. The plot shows clearly that there were three small studies with significant effects despite having a large standard error (right side). There were no studies with a similar standard error that were not significant (left side). Hypothetically, “imputing” the missing studies in the lower left corner of the plot would have increased the symmetry, and these studies would lie in the non-significance region of the plot or they would have a significant negative effect. In the upper side of the plot, there were large studies, some of which presented significant results of *p* < 0.05 or *p* < 0.10, and the distribution of effects is less asymmetrical.

The funnel plot seemed to be asymmetrical, and this was confirmed by Begg’s test (*p* = 0.016) (Figure 3). The contour-enhanced funnel plot presents three shaded areas based on the significance levels (<0.01, <0.05, <0.1). The plot clearly indicates that there were three small studies with significant effects despite having a large standard error. There were no studies with a similar standard error that were not significant. Hypothetically, “imputing” the missing studies in the lower left corner of the plot would increase the symmetry, and consequently, these studies would lie in the non-significance region of the plot, or they would have a significant negative effect. In the upper side of the plot, there were large studies, some of which presented significant results of *p* < 0.05 or *p* < 0.10, and the distribution of effects is less asymmetrical. In conclusion, the inspection of the plot suggested that the asymmetry may be caused by publication bias.

#### 3.4.2. People with OSAS Reporting Car Accidents versus People with OSAS without Car Accidents

Data were extracted from 10 studies [23,28,42,49,51,54,55,59,60,61].

**Figure 4:**
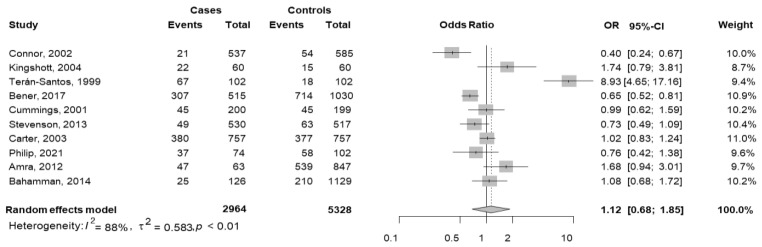
The forest plot of the subgroup meta-analysis of car accidents and non-car accidents with OSAS based on the questionnaire used [23,28,42,49,51,54,55,59,60,61]. Events Cases are defined as car accident with OSAS (total of 1000) and Total Cases as car accidents (2964); Events Controls are defined as non-car accidents with OSAS (total 2093) and Total Controls as non-car accidents (5328). In each group, a diamond represents the pooled OR estimate based on the random effects model.

In this analysis, the cases were defined as car accidents with OSAS (total of 1000) over the total car accidents (2964), and the controls were defined as non-car accidents with OSAS (total 2093) over total non-car accidents (5328). The pooled OR was equal to 1.12 (95% CI 0.68–1.85; *p* = 0.648), but the effect was not significant. The heterogeneity between studies was high (I^2^ = 88.1%, *p* < 0.001). Indeed, one study [51] reported a positive significant association, and two [42,54] reported significant negative associations (Figure 4).

Considering the study design, 2 studies were cross-sectional [23,28] and 8 were case–control [42,49,51,54,55,59,60,61]. In each group, the pooled OR was not significant (cross-sectional pooled OR = 2.08, 95% CI 0.59–7.39; case–control pooled OR = 1.08, 95% CI 0.57–2.04). No difference between the two groups was observed (*p* = 0.363); within each subgroup, the variability was high and significant (*p* < 0.001).

**Figure 5:**
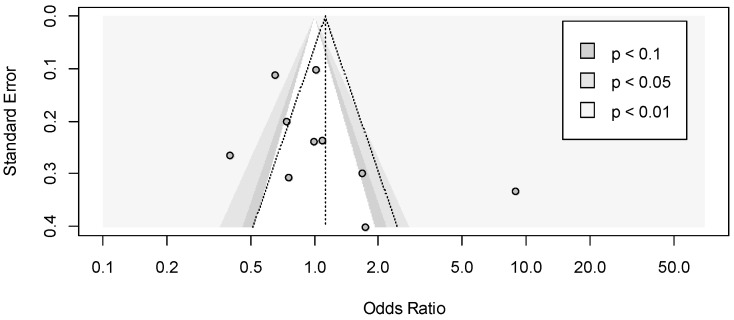
The contour funnel plot of the meta-analyses of car accidents and non-car accidents with OSAS, with contours of statistical significance at 1%, 5% and 10%. It is helpful to investigate if in a meta-analysis, some studies could have been suppressed based on their statistical significance. This representation facilitates the assessment of whether the areas where studies exist are areas of statistical significance and whether the areas where studies are potentially missing correspond to areas of low statistical significance. The funnel plot does not seem to be asymmetrical, and there seems to be a balanced distribution of studies based on the significances of their results.

The funnel plot did not seem to be asymmetrical, and this was confirmed by Begg’s test (*p* = 0.211) (Figure 5).

**Figure 6:**
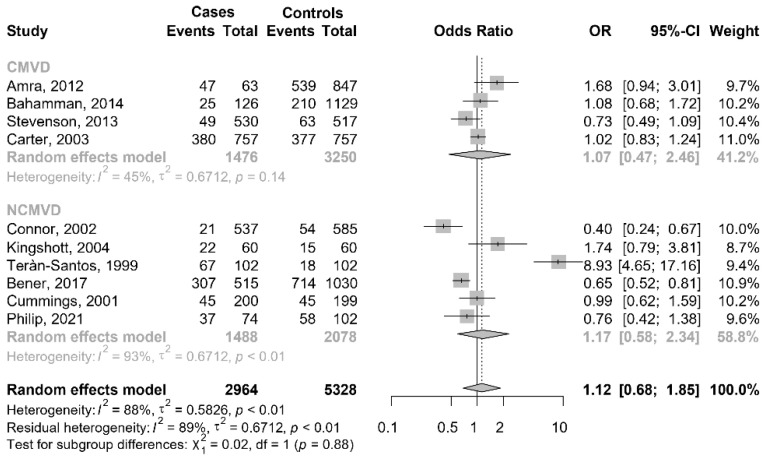
The forest plot of the subgroup meta-analysis of car accidents and non-car accidents with OSAS based on types of drivers [23,28,42,49,51,54,55,59,60,61]. Events Cases are defined as car accidents with OSAS (total of 1000) and Total Cases as car accidents (2964); Events Controls are defined as non-car accidents with OSAS (total 2093) and Total Controls as non-car accidents (5328). In each group, a diamond represents the pooled OR estimate based on the random effects model.

Based on the types of drivers we performed a subgroup meta-analysis. Four studies [23,28,59,60] considered CMVD drivers, and the remaining six [42,49,51,54,55,61] NCMVD drivers. In the first group, the result was not significant; the pooled OR was equal to 1.07 (95% CI 0.47–2.46), and the heterogeneity between studies in this group was not significant (I^2^ = 45%; *p* = 0.14). In the second group, the result was also not significant; the pooled OR was equal to 1.17 (95% CI 0.58–2.34), and the heterogeneity between studies in this group was high and significant (I^2^ = 93%; *p* < 0.001). Between the two groups, there was not a significant difference (*p* = 0.88) (Figure 6).

#### 3.4.3. People Reporting Car Crashes with ESS ≥ 10 versus People Reporting Car Crashes without ESS ≥ 10

Data were extracted from four studies [27,31,37,58]. In this analysis, the cases were defined as car crashes with ESS ≥ 10 (total of 1233) over total subjects with ESS ≥ 10 (3208), and the controls were defined as car crashes without ESS ≥ 10 (total 3000) over total subjects without ESS ≥ 10 (8517).

**Figure 7:**
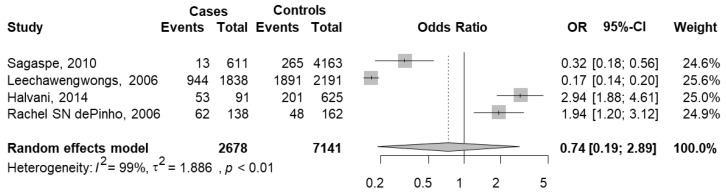
The forest plot of the meta-analysis of ESS ≥ 10 and car crashes [27,31,37,58]. Events Cases are defined as car crashes with ESS ≥ 10 (total of 1062) and Total Cases as subjects with ESS ≥ 10 (2678); Events Controls are defined as car crashes without ESS ≥ 10 (total 2405) and Total Controls as subjects without ESS ≥ 10 (7141). The whiskers represent the 95% confidence interval (CI). The diamond represents the pooled OR estimate based on the random effects model, with the center representing the point estimate and the width the associated 95% CI. I^2^ describes the percentage of the variability in effect estimates that is due to heterogeneity rather than sampling error, ranging from 0 (no heterogeneity) to 100 (maximum heterogeneity).

The meta-analysis results showed a pooled OR equal to 0.74 (95% CI 0.19–2.89; *p* = 0.6629), indicating that subjects with ESS ≥ 10 had a probability of having a crash that was 26% lower than that of controls, but it was not significant (Figure 7). The heterogeneity between studies was high (I^2^ = 98.6%, *p* < 0.001). Two studies [31,58] reported a significant positive association, and two others [27,37] showed a significant negative association.

Considering the study design, one study was case control [58], one was a self-answered questionnaire study [37] and the remaining two studies were cross-sectional [27,31]. Combining these three studies, the pooled OR was equal to 0.79 (95% CI 0.14–4.63), indicating a nonsignificant association. The within-group heterogeneity was significantly high (I^2^ = 96%; *p* < 0.001). These two studies reported opposite results.

Begg’s test was not performed because of the number of studies was fewer than 10. The evaluation of asymmetry in the funnel plot was difficult because of the small number of studies.

**Figure 8:**
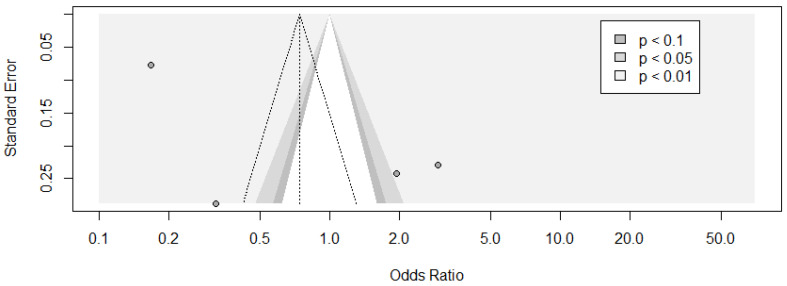
The contour funnel plot of the meta-analysis of ESS ≥ 10 and car crashes with contours of statistical significance at 1%, 5% and 10%. It is helpful to investigate if in a meta-analysis, some studies might have been suppressed based on their statistical significance. This representation facilitates the assessment of whether the areas where studies exist are areas of statistical significance and whether the areas where studies are potentially missing correspond to areas of low statistical significance. Three small studies with positive significant effects and one with a negative significant result despite having a large standard error were observed. One study with a large standard error reported a nonsignificant positive result. In the upper side of the plot, there was only a large study presenting a result with *p* < 0.01.

Two small studies with positive significant effects and one with negative significant result, despite having a large standard error, were observed. One study with large standard error reported a nonsignificant result. In the upper side of the plot, there was only one large study presenting a result with *p* < 0.01 (Figure 8).

#### 3.4.4. People Reporting Car Crashes with SDB versus People Reporting Car Crashes without SDB

Data were extracted from two studies [33,57]. The cases were defined as car crashes with SDB (total of 311) over total subjects with SDB (total of 513), and the controls were defined as car crashes without SDB (total of 264) over total subjects without SDB (total of 573).

**Figure 9:**
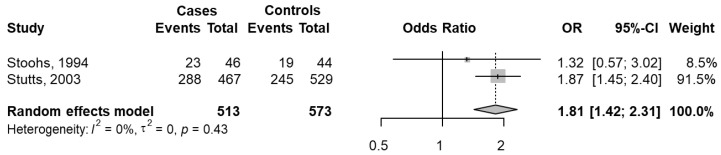
The forest plot of the meta-analysis of sleep-disordered breathing (SDB) and car crashes [33,57]. Events Cases are defined as total car crashes in subjects with SDB (total 311), and Total Cases indicate Total subjects with SDB (total 513). Controls Cases are defined as car crashes in subjects without SDB (total 264), and Total Cases indicate total subjects without SDB (total 573). The whiskers represent the 95% confidence interval (CI). The diamond represents the pooled OR estimate based on the random effects model, with the center representing the point estimate and the width the associated 95% CI. I^2^ describes the percentage of the variability in effect estimates that is due to heterogeneity rather than sampling error, ranging from 0 (no heterogeneity) to 100 (maximum heterogeneity).

The pooled OR was significant and equal to 1.81 (95% CI 1.42–2.31; *p* < 0.001), indicating that for subjects with SDB, the probability of having a car crash was 1.81 times that of subjects without SDB. The heterogeneity was not significant (I^2^ = 0%; *p* = 0.431) (Figure 9).

### 3.5. Gender-Based Analysis on Study Population

The qualitative analysis covered a total of 49 analyzed studies. Only 26 of them provided data on the percentage of women in the enrolled population. Female participation ranged from a minimum of 0.5% in the Ebrahimi study [26] to a maximum of 61.3% in the Powell study [32], with an average of 25%. Year of publication of the studies considered in the qualitative analysis and ranged from the earliest in 1987 to the most recent in 2021.

As gender medicine is a relatively new branch of medicine and we were trying to explain the low percentage of women in the recruited populations, studies published after 2010 were analyzed (18 out of 26). These later studies found an average female proportion in the enrolled population of 24.68 %. Of all the studies that reported the proportion of women in the study population, only one study reported epidemiological data on OSAS and road accidents in the female population. [40]

Finally, 48 out of 49 studies lacked results on the risk of road accidents in the population of adult women who drive a road vehicle and have risk factors such as OSAS or sleepiness. For this reason, it was not possible to perform a meta-analysis with a gender-based approach.

## 4. Discussion

The purpose of this systematic review was to analyze the correlations between OSAS, daytime sleepiness and the likelihood of road accidents in adult working populations. Moreover, the aim was to separately analyze the epidemiology of the investigated phenomenon in populations of adult working women versus men.

High heterogeneity was found among the studies included in the meta-analysis: different subgroup classifications were necessary as the definition of cases and controls differed from one study to another.

In order to be as inclusive as possible, the classifications used in the analyzed studies differed in the definitions of cases and controls. In the included studies, cases were defined as: (i) car crashes in subjects with OSAS and total number of subjects with OSAS; (ii) car accidents in subjects with OSAS and total number of car accidents; (iii) car crashes in subjects with ESS ≥10 and total subjects with ESS ≥10; (iv) car crashes in subjects with SDB and total subjects with SDB. Additionally, the first two definitions of cases were analyzed depending on the different types of drivers (CMVD, commercial motor vehicle drivers and NCMVD, non-commercial motor vehicle drivers).

This variability was due to the different designs and settings of the studies. In fact, some of them were run in sleep clinics using PSG-mediated diagnosis or were instead focused on traffic accidents by accessing traffic databases, while for the most part, the road accidents were self-reported within specific time frames that varied between surveys.

Only one case–control study, from 1989 [40], included epidemiological data on road accidents in OSAS patients of both sexes. Based on the results of this systematic review, the gender-based approach is completely absent in studies that report data on road accidents in patients with OSAS or daytime sleepiness. Ward et al. suggested that differences between men and women are attributable to driving exposure that may act as a cofounding factor because more men than women are professional drivers and to a higher degree of self-awareness in women who self-regulate their car crash risk by avoiding driving [38].

Not all the analyzed studies accurately reported the characteristics of the enrolled subjects: however, in 13 studies, they were professional drivers (truck and/or bus drivers) [22,23,25,26,28,31,33,37,58,59,60,62,66]; in 22 studies, they were laypeople, i.e., non-professional drivers defined as licensed adult drivers [27,29,30,32,34,35,36,38,46,47,49,50,51,52,53,55,56,61,65,67,69,70]; 1 study concerned firefighters [23]; in 5 cases, they were patients from sleep laboratories [40,43,44,45,68] and in the final 2 cases, they were patients who were hospitalized after road accidents [54,57].

These can be associated with different types of driving and different numbers of kilometers traveled during the year, and it is certainly difficult to imagine that data on professional drivers can be superimposed onto laypeople. Moreover, since most professional drivers are male, there may be a shift in the interpretation of this phenomenon, leading to imprecise conclusions about the prevalence of OSAS in the male and female populations.

Garbarino et al. [72] showed in their systematic review that OSAS is an underdiagnosed condition and that there is a nearly twofold risk of work accidents and occupational driving crash in subjects with OSAS compared with those without. The result of the present meta-analysis showed a stronger correlation between instrumentally diagnosed OSAS and the risk of car crashes (OR 2.36; 95% CI: 1.92–2.91).

A very recent systematic review of 14 articles by Moradi et al. [73] showed a significant association between crash involvement and sleepiness and that drowsy driving increased road crashes by 1.29 to 1.34 times compared with driving without sleepiness [73]. These findings are in contrast with the results of the current meta-analysis, which indicated a nonsignificant association between subjects with ESS ≥ 10 and the probability of having a car crash. These conflicting results deserve some explanations. Moradi et al. [73] included studies with different definitions of sleepiness (sleep disorders, sleep deprivation, sleep fragmentation, daytime sleepiness, insomnia, snoring, sleep quality and quantity), while the current review assessed the correlation between subjects reporting an ESS ≥ 10 and car crash experience.

The correlation between sleepiness and the risk of road accidents has long been studied, and the results were often conflicting [74,75]. Ward et al., in their retrospective case-series observational study, showed strong correlations between sleepiness at the wheel, near misses and actual accidents [38]. Therefore, sleepiness may be correlated with the risk of road accidents, while it may not be correlated with the AHI-defined severity of OSAS. In fact, Ward et al. found a correlation between near misses and related accidents and somnolence, but not with the severity of OSAS expressed in AHI; they did, however, show that subjects enrolled in the sleep laboratory with untreated OSAS, (AHI > 5) were three times more likely to report a road accident than the general population [38].

By contrast, the review by Bioulac et al. showed a nonsignificant association between daytime sleepiness as quantified by ESS and accident risk [76]. The authors underlined that evaluation by ESS and evaluation of sleepiness at the wheel do not concern the same type of sleepiness and that sleepiness at the wheel should be systematically added to ESS when investigating fitness to drive [76].

Questionnaire studies conducted in a work setting might be subject to underreporting from workers aiming to avoid the diagnosis. Therefore, the preferred approach should be a clinical and instrumental evaluation with the help of validated questionnaires by a physician skilled in the management of patients with OSAS.

Regarding the results for the female population, it was not possible to perform a meta-analysis of the correlated risk in adult working women driving a road vehicle between the presence of OSAS and/or somnolence and the possibility of a driving accident: of the 49 articles that were qualitatively evaluated, only 26 reported data on the percentage of the female population recruited in clinical trials. The average percentage was 25%, and it ranged from a minimum of 0.5% to a maximum of 61.3%. To the great surprise of the authors, these studies showed the proportion of women enrolled in the studies, but when presenting the results for the presence of OSAS and sleepiness in relation to the number of road accidents, the female population was no longer separate but was aggregated with the male majority. In order to verify whether the low representation of the female population was mainly due to the outdated trial publication dates, we analyzed the number of trials published after 2010. However, in these 18 studies, the percentage of the female population in the entire study group was even lower (24.48%).

This evidence only supports doubts about the methodologies used in recruiting patients and strengthens confidence that gender medicine seeks to prove its principles even in studies conducted in the last ten years.

There is no doubt that there is a great need for rigid rules to integrate gender medicine into clinical trials in all branches of medicine.

## 5. Conclusions

The present systematic review has some limitations. Weaknesses and heterogeneities of studies can undoubtedly reduce the power of statistical association quantified in this study. In fact, variability in how OSAS and sleepiness were defined, and quantified, varying populations assessed in the trials (commercial and non-commercial drivers, laypeople, sleep lab patients with driving licenses), differences in epidemiological designs and in car crash reporting (i.e., the majority of cases were self-reported, and only a few were found in police recordings) and different observation periods. Again, a possible source of bias in this meta-analysis is the different work organizations between workers with and without night shift work, which implies sleep–wake cycle disruption and sleep deprivation, and this could be a variable that changes the relationship between OSAS and driving accidents.

The lack of well-conducted studies with a gender-based approach is impressive, considering that so many people of both sexes drive their car both to work and during work. The main reason is that OSAS is still not considered a risk factor pertaining to work. Moreover, in Europe OSAS is not yet recognized as a clinical disease linked to driving risk.

In summary, the best available evidence suggests that people with OSAS have an OR of 2.36 of risk of a motor vehicle crash compared with the general population. No significant correlation has been found between sleepiness and car crashes. SDB was significantly correlated with the risk of motor vehicle crashes.

In conclusion, sleep disorders should be systematically considered when investigating fitness to drive both in laypeople and in professional drivers. Road safety programs and epidemiological screenings in both female and male populations are needed along with programs to inform drivers and communities about the existing risk.

## Figures and Tables

**Table 1:** Newcastle–Ottawa Scale adapted for cross-sectional studies.

Author		[22]	[23]	[24]	[25]	[26]	[27]	[28]	[29]	[30]	[31]	[32]	[33]	[34]	[35]	[36]	[37]	[38]
**Selection**: (Maximum 5 stars)	(1) Representativeness of the sample	*		*	*	*	*		*	*	*	*			*	*	*	
	(2) Sample size	*	*	*	*	*	*		*	*	*	*			*	*	*	*
	(3) Non-respondents	*				*		*	*	*		*	*	*	*		*	
	(4) Ascertainment of the exposure (risk factor)	*	*	**	*	**	*	*		*	**	**	**	**	*	*	**	*
**Comparability**: (Maximum 2 stars)	(5) The subjects in different outcome groups are comparable, based on the study design or analysis. Confounding factors are controlled.	*	*	*	*	*	*	*	*	*	*	*	*	*	*	*	*	*
**Outcome**: (Maximum 3 stars)	(6) Assessment of the outcome	*	*	*	*	*	*	*	*	*	*	*	**	**	*	*	*	*
	(7) Statistical test	**	*	*			*	*	*	*	*	*	*	*	*	*	*	*
Total score=		8	5	7	5	7	6	5	6	7	7	8	7	7	7	6	8	5

* The tool is available or described; ** Validated measurement tool.

**Table 2:** Newcastle–Ottawa quality assessment scale: case–control studies.

Author		[39]	[40]	[41]	[42]	[43]	[44]	[45]	[46]	[47]	[48]	[49]	[50]	[51]	[52]	[53]	[54]	[55]	[56]	[57]	[58]	[59]	[60]	[61]	[62]	[63]
**Selection**: (Maximum 4 stars)	(1) Is the case definition adequate?	*	*	*		*	*		*		*		*	*		*	*				*	*		*		
	(2) Representativeness of the cases	*	*	*	*	*	*	*	*		*	*	*	*	*	*	*	*	*	*	*	*	*	*		*
	(3) Selection of controls	*	*	*	*	*		*	*			*	*			*	*	*	*	*		*		*	*	*
	(4) Definition of controls	*	*	*	*	*		*	*	*	*	*	*	*	*	*	*	*	*	*	*	*		*	*	*
**Comparability**: (Maximum 2 stars)	(5) Comparability of cases and controls on the basis of the design or analysis	*	*	*	*	*	*	*	*	*	*	*	*	*	*	*	*	*	*	*	*	*	*	*	*	*
**Outcome**: (Maximum 3 stars)	(6) Ascertainment of exposure						*		*		*	*	*	*	*	*							*			
	(7) Same method of ascertainment for cases and controls	*		*	*	*	*		*	*	*	*	*	*	*	*	*	*		*	*	*	*	*	*	*
	(8) Non-response rate	*		*						*				*	*						*			*		
Total score=		6	5	7	5	6	5	4	7	4	6	6	7	7	5	7	6	5	4	5	6	6	4	7	4	5

* The tool is available or described.

**Table 3:** Newcastle–Ottawa quality assessment scale: cohort studies.

Author		[64]	[65]	[66]	[67]	[68]	[69]	[70]
**Selection**: (Maximum 4 stars)	(1) Representativeness of the exposed cohort	*	*	*	*	*	*	*
	(2) Selection of the non-exposed cohort		*	*	*	*	*	*
	(3) Ascertainment of exposure	*						
	(4) Demonstration that outcome of interest was not present at start of study				*			*
**Comparability**: (Maximum 2 stars)	(5) Comparability of cohorts on the basis of the design or analysis	*	*	*	*	*		*
**Outcome**: (Maximum 3 stars)	(6) Assessment of outcome	*	*	*	*	*	*	*
	(7) Was follow-up long enough for outcomes to occur	*	*	*	*	*		
	(8) Adequacy of follow up of cohorts			*	*			
Total score=		5	5	6	7	5	3	5

* The tool is available or described.

## Data Availability

Not applicable.

## Supplementary Materials

The following supporting information can be downloaded at: https://www.mdpi.com/article/10.3390/jcm11143971/s1, Table S1: Characteristics of the included studies.
